# Shrew Communities in Mediterranean Agro-Ecosystems of Central Greece: Associations with Crop Types, Land Uses, and Soil Parameters

**DOI:** 10.3390/life13122248

**Published:** 2023-11-23

**Authors:** Vasileios Bontzorlos

**Affiliations:** Civil Society Organization TYTO—Association for the Management and Conservation of Biodiversity in Agricultural Ecosystems, 41335 Larisa, Greece; info@tyto.org.com or vasilibon@gmail.com; Tel.: +30-6946025339

**Keywords:** shrews, agriculture, Thessaly, Barn owl, multivariate analysis

## Abstract

Shrew communities play a crucial role in a diverse range of natural, urban, and agricultural ecosystems. We used Barn owl diet analysis as the ideal proxy to assess small-mammal distribution patterns on large spatial scales. More than 10,000 pellets were analyzed from Thessaly, the largest agricultural prefecture located in central Greece. A total of more than 29,000 prey items were identified, one of the largest datasets used in similar analyses in Europe. Three discrete shrew species were present in Thessaly agricultural plains, central Greece (Güldenstädt’s shrew *Crocidura gueldenstaedtii*, Bi-coloured shrew *Crocidura leucodon*, and Pygmy white-toothed shrew *Suncus etruscus*), which comprised a total of 7452 shrews, representing 25.64% of the total small-mammals’ dataset. *C. gueldenstaedtii* and *S. etruscus* demonstrated strong associations with heavy argillaceous-clay soils and Vertisol soil types, whereas *S. etruscus* was also associated with non-irrigated land and non-intensive cultivated plots. *C. leucodon* demonstrated no significant associations to any environmental gradient and demonstrated habitat plasticity, most possibly shaped by existing resources and competition. Our study highlights the important insights gained from Barn owl diet analysis in respect of small-mammal assemblages on broad geographical scales, and the inclusion of soil parameters as drivers of habitat suitability and distribution patterns for small-mammal responses.

## 1. Introduction

Shrew communities are crucial components of natural, semi-natural, urban, and peri-urban ecosystems around the world, including a diverse range of agroecosystems as well [1,2,3]. Within the mammalian group, shrews along with rodents and bats comprise the most diverse orders globally [4]. Due to their small home ranges, limited life-span, and complex ecological niches, it is a difficult and demanding process to delineate the underlying mechanisms driving shrew associations with habitat types, habitat management, and land uses, especially on broad geographical scales [1,5,6].

Furthermore, during recent decades, agricultural extensification and intensification have been the main drivers behind habitat modifications and have been recorded as primary threats for biodiversity in lowlands all over Europe and globally [7,8,9,10]. As such, the delineation of biological organisms’ home ranges, population abundances, and their associations with environmental and ecological parameters is of major importance specifically in continuously changing agro-ecosystems. Modeling how small-mammal species respond is needed to comprehend the requirements of biodiversity conservation in the future and to deal with upcoming challenges in agricultural practices [11,12].

**Figure 1:**
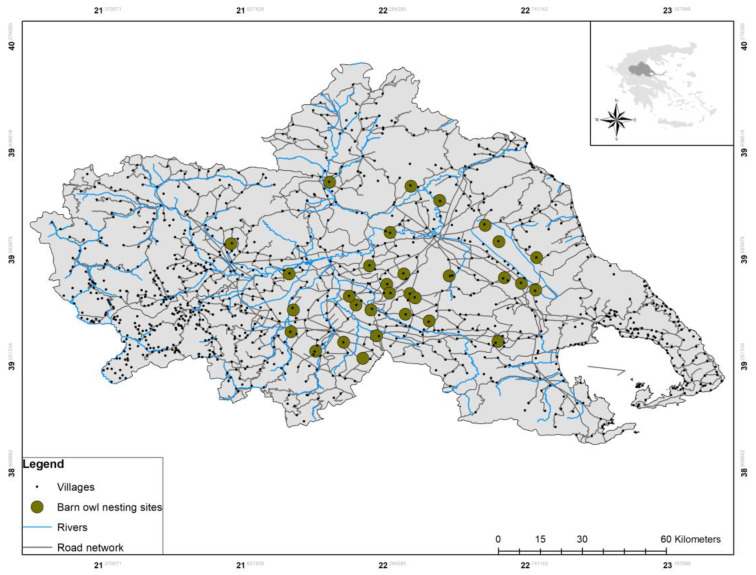
Study area located in Thessaly plains, the largest agricultural ecosystem of Greece. Dark green circles indicate the 31 natural Barn owl breeding sites in which pellets were collected. Total area of agricultural plains approximately at 5000 sq. km.

Considering that agriculture is currently the dominant use of land and the major driver of environmental change that shapes agricultural landscapes [13], our study was conducted in the largest and most extensively managed agroecosystem of Greece, covering approximately 5000 sq. km. of the Thessaly plains [6] (Figure 1). Shrew associations with habitat and land uses have never been studied previously on large spatial scales in Greece, and it is the first time they are assessed in the Mediterranean agro-ecosystems of central Greece.

Shrews as small-size mammals are especially prone to global change powers such as climate change and land use change, which shape food, habitat, and predation and may alter the community dynamics of small mammals. Especially, land use change in open Mediterranean habitats can be an important threat to the resilience of shrew communities [14,15]. In addition, shrews play a very important role in the functioning of ecosystems both as predators and prey [16]. In order to meet both challenges—(i) to explore the interactions between shrew populations and crop types, land uses, and habitat characteristics, and (ii) to delineate distribution patterns and responses to environmental gradients—on a large spatial scale in the intensive agroecosystems of Thessaly plains, we assessed shrew populations through the spectrum of Barn owl diet analysis, as previously performed for voles in the same region [6]. Through the diet of the Barn owl, we can establish first-level knowledge of the association of shrews to specific underlying environmental parameters in the agricultural landscape and define the areas where each different species is present with higher abundances [6].

Barn owl trophic analyses are a much more effective alternative to monitor and estimate small-mammal populations in respect of small-mammal trapping [3]. The Barn owl diet can function as an ideal solution to assess the distribution, composition, and abundance patterns of small mammals on broad geographical scales [17,18], and Barn owl pellet analyses have been repeatedly used in the scientific literature as a proxy [6,19,20,21,22,23,24,25,26]. The methodology involves bisecting the pellets and extracting the skulls, mandibles, femur, pelvis girdles, etc. In continuation, visual identification with the use of key-books and measurements takes place, and each recovered prey item is identified to the species level. The limitations of this method lie in the following facts: (i) pellet analysis and prey identification are very time-consuming processes, and (ii) soil types and land uses possibly associate with specific vegetation types that attract specific small-mammal species, thus creating a complex rather than a straight-forward effect when associations are explored with small-mammal species. This can be dealt with up to a point, by including both aspects (e.g., land uses and soil types) in the predictor datasets.

## 2. Materials and Methods

### 2.1. Field Work and Data Collection

Field monitoring was realized and data were collected according to Bontzorlos et al. [6]. From a total of 31 high-fidelity Barn owl natural breeding sites in Thessaly plains, four seasonal pellet samplings were realized from 2003 to 2005 in each breeding site representing two breeding periods (April to August) and two non-breeding periods (October to February). A total of 10,065 Barn owl pellets were analyzed using the dry method [27,28], whereas all skulls and bones that were recovered were then identified based on measurements and morphology [29,30,31,32,33,34,35,36,37].

### 2.2. Data Analysis

In order to explore the response of shrew communities and changes in their community composition upon the recorded environmental gradients, ordination techniques were applied, with the software Canoco 5.0 for Windows, Microcomputer Power, Ithaca, New York, USA [35,36]. Principal Component Analysis (PCA) was primarily applied to the matrix of response variables in the form of an indirect gradient analysis, in order to assess whether linear or unimodal methods must be used; redundancy analysis (RDA) was then applied to both datasets of response and predictor variables. The constrained ordination (RDA) actually creates new two-dimensional axes from the multidimensional space occupied by the predictor variables (environmental gradients), which correspond and summarize the greatest variability in response variables within the datasets that can be best explained by the environmental variables [38,39].

Before applying further data analysis, the predictor variables were tested for possible collinearity effects by calculating the Variance Inflation Factor (VIF). VIFs were calculated for each predictor as the inverse of the coefficient of non-determination for a regression of that predictor on all others and were actually positive values, which represent the overall correlation of each predictor with all others in a model. The VIF is a measure of the amount of multicollinearity that exists when there is a correlation between independent variables in a multiple regression model, which can adversely affect the results [40,41,42].

Before removing highly collinear variables from the predictor dataset, and then choosing those predictor variables to be introduced in analysis through a forward selection process, a permutation test on the constrained ordination model using all the considered explanatory variables was implemented [39]. When their joint effect was demonstrated to be significant, we proceeded with the forward selection process that shaped the final predictor dataset of the model. The contribution of each independent variable was also calculated through a forward selection process.

The responses of each different shrew species were tested upon each environmental gradient with the application of a Linear Model (Generalized Linear Model with Gaussian distribution) using the Canoco software, and the best-fit model was finally chosen with the use of the Akaike criterion (AIC). Shrew abundance in Thessaly was graphically visualized through the use of the inverse distance weighting interpolation method [43,44] and shrews’ response curves upon environmental gradients were produced in Canoco 5 [38,39].

### 2.3. Datasets

In order to create a response variable matrix, for each different species, the relative frequency of identified prey items was included per season (4 seasons) and each different breeding site (31 sites), giving a total of 124 rows (4 seasons × 31 sites). In respect of columns, each column comprised each different small mammal, including the species’ relative frequency per cell. Similarly, for the construction of the predictor dataset, within a radius of 2 km around each Barn owl breeding site, we calculated the area occupied by each predictor variable. Predictor variables are presented in Table 1.

Land use variables, soil variables, and crop types were calculated by combining the following: (i) agricultural datasets were provided by the statistical service offices in each different region of the Thessaly prefecture; (ii) 1:5000 maps were delivered by topographic services in each respective regional office of the Thessaly prefecture; (iii) in situ GPS point verification; and (iv) 1:20,000 maps of soil cartography were specifically provided by the Institute of Cartography and Soil Taxonomy of the Hellenic Agricultural Organization Demeter and [1,6].

## 3. Results

From the total of 15 small-mammal species identified through Barn owl trophic analysis in Thessaly plains, 3 different shrew species were recorded in the agricultural ecosystems of central Greece: Bicoloured shrew *C. leucodon*, Güldenstädt’s shrew *C. gueldenstaedtii*, and the Pygmy white-toothed shrew *S. etruscus*. In total, 7452 shrews were preyed, representing 25.64% of the total prey intake in the Barn owl diet in terms of relative frequency, but only 5.64% in terms of biomass due to the small size of shrews. Shrews were the second-most abundant small-mammal group in Thessaly following the primary abundant voles’ group (11,770 vole prey items, 40.50% relative frequency). *C. gueldenstaedtii* was the most abundant shrew species in Thessaly plains (21.43%) and the second-most abundant species out of 15 small-mammal species. The other two shrew species followed with smaller abundances, *C. leucodon* with 2.44% and *S. etruscus* with 1.64%. *C. gueldenstaedtii* was present at all 31 sampling sites, *C. leucodon* was present at 28 sites, and *S. etruscus* was recorded present in 27 sites.

Once the highly collinear variables with VIF > 10 were removed and forward selection included the most important predictor variables in the model, both predictor and environmental datasets were introduced for ordination analysis. The indirect gradient analysis was applied to the response matrix, and PCA demonstrated that linear methods should be used henceforth to produce the constrained model, since the largest gradient’s value was less than 3 (2.0 SD units long) (Table 2). Therefore, a direct-gradient redundancy analysis (RDA), or constrained analysis, was applied to both the response and predictor matrices. All produced canonical (constrained) axes were measured as the percentage of the explained variance and permutation, resulting in a significant constrained model (first axis: pseudo-F = 2.1, *p* = 0.0099; all axes: pseudo-F = 4.4, *p* = 0.0099). This suggested that the constrained environmental axis could explain the variability within the response matrix, where the first two constrained axes explained almost 85% of the fitted variability in the response dataset (Table 2).

**Figure 2:**
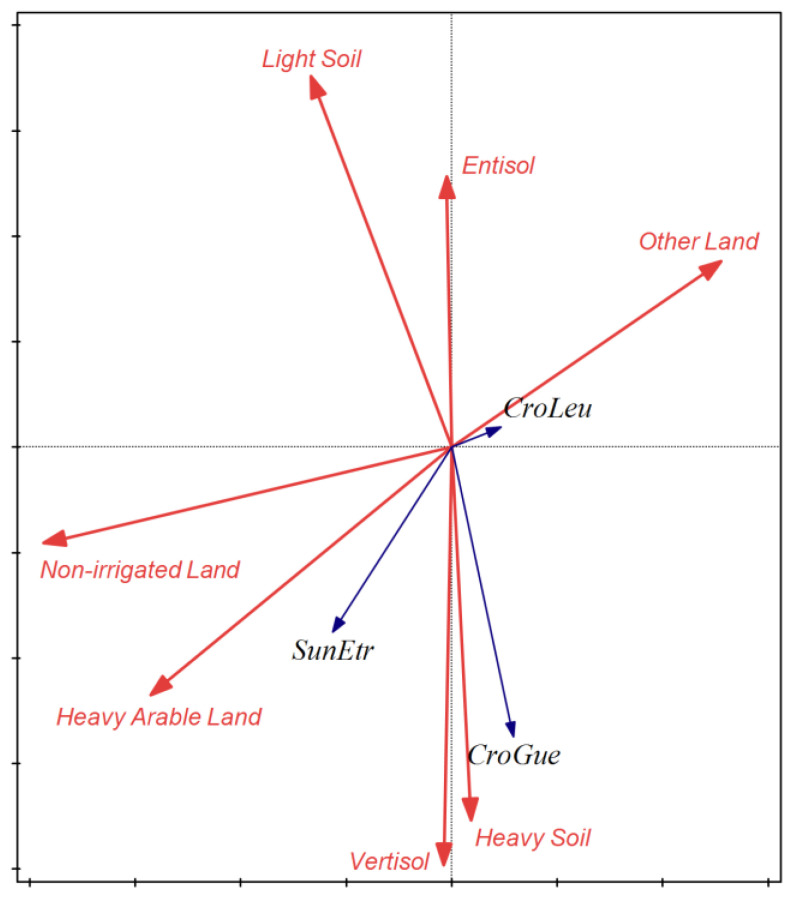
Biplot of constrained ordination produced from RDA upon the matrices of shrew species recorded in 31 sites of Thessaly plains through the Barn owl diet spectrum (response variables matrix) and the predictor variables recorded in each site/plot spot (environmental variables matrix). The graph is a two-dimensional representation of the variability in shrew species presence and abundance, which is explained by the variance in the environmental gradients defining each site. Blue vectors represent shrew species, and red vectors represent environmental variables. Clustering of shrew species in different quadrants indicates a difference in the attachment and associations of shrews to land uses, crops, and soil characteristics, and the adjacency of shrew vectors to environmental vectors denotes which environmental variables mainly affect the presence of each animal in each case. CroLeu: Bicolored shrew (*Crocidura leucodon*), CroGue: Güldenstädt’s shrew (*Crocidura gueldenstaedtii*), SunEtr: Pygmy white-toothed shrew shrew (*Suncus etruscus*), Non-irrigated Land: remaining area after extracting irrigated land from the total agricultural land in each site, Heavy Arable Land: Cereal crops (wheat, barley, oat) and Industrial Cultivations (dominated by cotton, and to lesser extent tobacco and sugar beets), Vertisol: vertisol soil type, Heavy Soil: Argillaceous-Clay soil texture, Other Land: Fallow Land, Hills, Grassland, Pastures and Urban Areas, Entisol, Light Soil: Sandy-Clay soil texture.

A forward selection process was then applied to the predictor dataset, using partial Monte Carlo permutation tests in order to assess the usefulness of each potential predictor, formulating a new subset of seven explanatory variables that comprised the final explanatory dataset (Table 3). The new datasets delivered a new significant constrained ordination model (first axis: pseudo-F = 3.5, *p* < 0.01; all axes: pseudo-F = 6.5, *p* < 0.01). The constrained ordination biplot that includes both the response and predictor variables is shown in Figure 2, which visualizes the RDA results of the constrained model, and depicts how the three shrew species are positioned in the ordinational space in relation to the environmental gradients that drive their responses.

**Figure 3:**
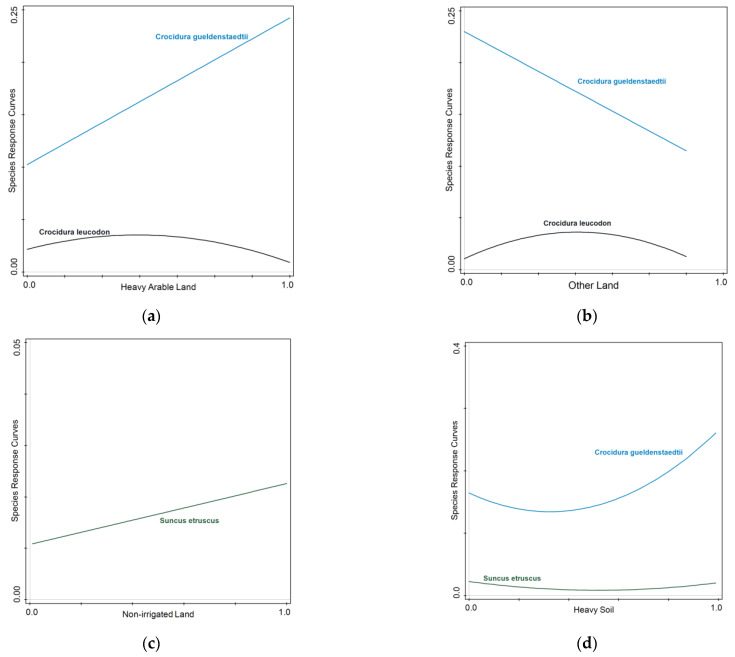

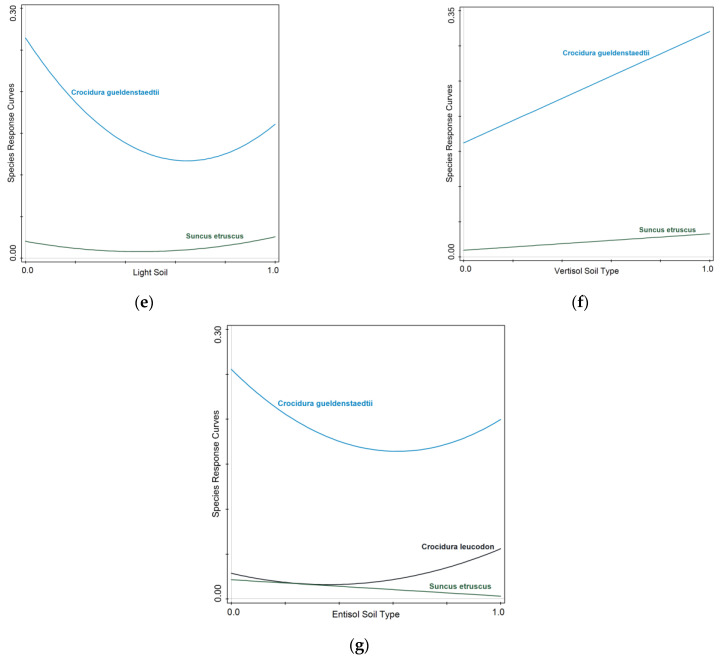
The response patterns of individual shrew species upon each different environmental variable are demonstrated. Responses that fitted a significant 1st- or 2nd-order polynomial model are included in the graphs. Shrew response curves are demonstrated upon the following environmental gradients: (**a**) “Heavy Arable Land”, (**b**) “Other Land”, (**c**) “Non-irrigated Land”, (**d**) “Heavy Soil”, (e) “Light Soil”, (**f**) “Vertisol Soil Type”, (**g**) “Entisol Soil Type”.

Each one of the three different shrew species in Thessaly plains demonstrated a different response to the studied environmental variables included in the model. The best-fit response model was selected as a 1st-order or a 2nd-order polynomial model based on a linear model analysis (Generalized Linear Model with Gaussian distribution) and the Akaike criterion for each shrew response to a different environmental gradient (Table 4). Furthermore, the discrete response models for each shrew species were visualized using the Canoco 5.0 for Windows, Microcomputer Power, Ithaca, New York, USA software, through the species response curves application (Figure 3).

**Figure 4:**
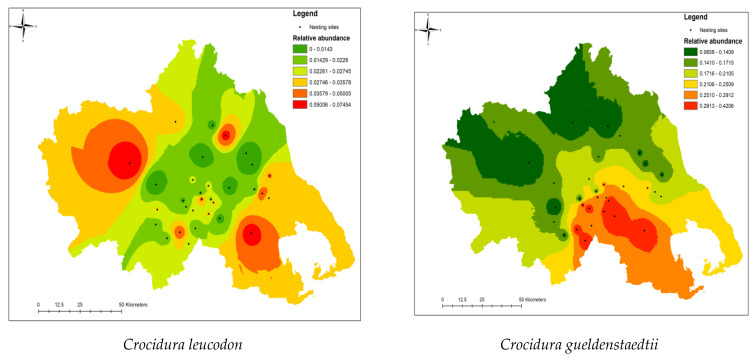

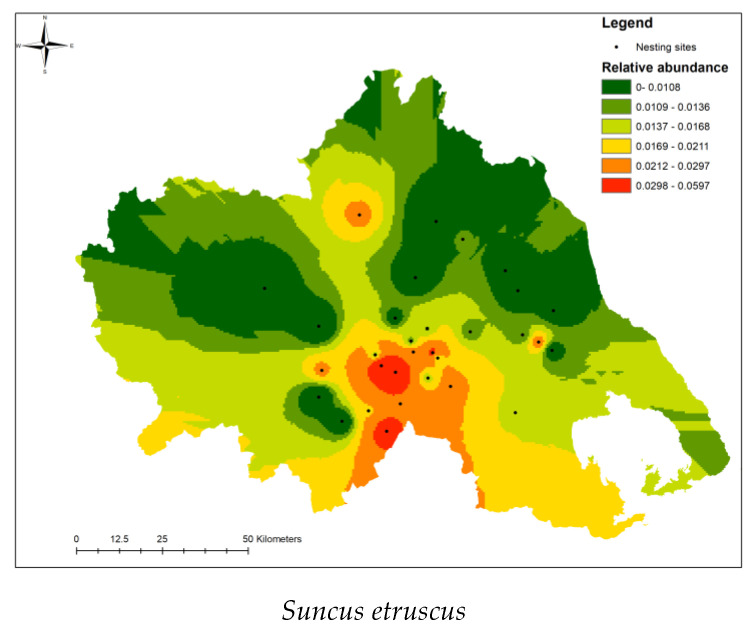
Distribution patterns of three shrew species (*C. leucodon, C. gueldenstaedtii, S. etruscus*) in the agricultural ecosystems of Thessaly, based on the spatial interpolation on relative frequency from 31 Barn owl breeding sites, where Barn owl pellet analysis was carried out. Interpolation was realized using inverse distance weight model (IDW). In order to determine cell values, a linear weighted combination of measured values from sample points was used, where the weight is a function of the inverse distance from the output cell location. *C. leucodon* distribution patterns in the upper left panel; *C. gueldenstaedtii* distribution patterns in the upper right panel; *S. etruscus* distribution patterns in the lower panel.

The discrete response models for each one of the three shrew species in Thessaly plains, *C. gueldenstaedtii*, *C. leucodon,* and *S. etruscus* (Table 4, Figure 3), are also geographically visualized through different abundance hot-spots and distribution patterns in the agroecosystems of Thessaly. We performed an interpolation using the Inverse Distance Weighted method (IDW) and the geostatistical analyst extension to ArcMap, in order to create shrew distribution maps and to pinpoint the different strongholds for each shrew within the agricultural ecosystem, which are depicted in the three subfigures of Figure 4.

## 4. Discussion

We used the Barn owl diet analysis in the agroecosystems of Thessaly, central Greece, as a proxy and assessed the small-mammal distribution and response to environmental gradients. Small mammals function as an ideal species group to formulate questions at different scales ranging from small plots to extensive landscapes [42,45,46]. Shrews were the second-most abundant small-mammal group in Thessaly plains. Although *C. gueldenstaedtii* was the most abundant shrew species (and the second-most abundant small mammal in the area) with much higher values than *C. leucodon* and *S. etruscus*, all shrew species were present in the majority of the 31 sampling sites, indicating a broad geographical presence and distribution range in Thessaly. Nonetheless, shrews demonstrated distinct responses upon different land uses, crops, and soil characteristics differentiating their distribution patterns and their abundance hotspots in the plains of central Greece.

*C. gueldenstaedtii*, as demonstrated in the constrained ordination biplot, indicated a clear attachment to soil characteristics and soil types, but not to agricultural land uses, crop types, or other types of land uses (Table 3, Figure 2 and Figure 3). The perpendicular positioning of the *C. gueldenstaedtii* vector to the predictor vectors that comprise cereal and industrial crops, non-irrigated cultivated land, and different types of land uses such as fallow land, grasslands, pastures, and urban areas indicate that the presence of *C. gueldenstaedtii* is clearly affected neither by any type of land uses nor crop types. *C. gueldenstaedtii* has been recorded to prefer habitats with dry terraces and dry ground in northern Europe [47,48], and a variety of habitats in the Mediterranean basin ranging from humid, wet, and dense vegetation cover to rocky, grasslands and shrublands, woodland and forest, agricultural mosaics, and even marine and coastal habitats [49,50]. In contrast to that, the species is found in a multitude of sampling sites comprising an agricultural mosaic of land uses and crop types, in other studies and in our study area as well. Our study is the first to our knowledge that the presence of *C. gueldenstaedtii* in Thessaly plains is primarily defined and attached to heavy argillaceous Vertisol soils, without any concrete attachment between *C. gueldenstaedtii* and specific land uses and crops. There have been studies in the past that have recorded the presence of shrew species in heavy clay soils [51,52,53,54,55], but none has depicted such a strong association on a large geographical scale as the one produced from our dataset. Heavy soils rich in clay, and Vertisol soils, are typically found on level or mildly sloping topography, and form deep wide cracks from the surface and downward when they dry out [6,56]. The presence of shrews in such “subterranean” habitats can be explained because they provide both shelter and a place where resources (seeds, insects, etc.) can accumulate [53]. Vertisol soil types and argillaceous-clay soil textures, which support abundant Güldenstädt’s shrew assemblages in Thessaly, also maintain higher levels of moisture and humidity than the rest of the soil types and soil texture of the study area [56], and therefore possibly meet that specific need of the species as well [49,50].

In a similar context, *S. etruscus* indicates a strong association with Vertisol soils as well, but in contrast to *C. gueldenstaedtii*, demonstrates an additional attachment to non-irrigated arable agricultural/cultivated land. That fact is also corroborated by the species’ individual response models (Table 4, Figure 3). *S. etruscus* is still an unknown species in parts of its range [57]. In Greece, it is considered to have a very scattered and scarce distribution, and it was considered to be completely absent from the plains of Thessaly, presenting only a very small population in a small southern mountainous region [57,58]. Nonetheless, data of this study indicated that the species was present in 27 of 31 sampled sites in Thessaly plains, proving the established presence of the species in the study area [59]. In Europe, *S. etruscus* is strictly attached to the Mediterranean basin and its climatic conditions. It mainly inhabits natural grasslands and open places with maquis vegetation; is also often encountered in vineyards, olive groves, and sometimes gardens; and avoids intensively cultivated land and dense forests [47,57,60,61,62]. In Thessaly as well, the increase in *S. etruscus* in non-irrigated land, which comprises agricultural plots without the intensified practices of deep tillage and heavy irrigation, corroborate these findings where the species avoids intensively cultivated plots.

The third representative of shrew communities in Thessaly plains was *C. leucodon*. In contradiction to *C. gueldenstaedtii* and *S. etruscus*, *C. leucodon* formed very weak associations with all predictor variables, thus demonstrating a very weak vector in Figure 2 close the center of the quadrants, a fact that denotes weak connections to the recorded explanatory dataset [38,39]. That finding was also verified with the individual response models, where *C. leucodon* presented, on one the hand, significant 2nd-order polynomial models (Table 4); but on the other hand, the response curve graphs demonstrated peaks in the middle of the X axis, denoting an actual no-preference for any specific type of land use or crop type, apart from a small increase toward Entisol soil types (Figure 3). The species has also been recorded in previous studies to have a certain habitat plasticity in Europe. In France, *C. leucodon* is found in damp areas with dense vegetation; in central Europe and Italy, it prefers open agricultural landscapes; at the northern edge of its range, it is associated with gardens and houses in suburban and urban areas; in the Balkans and Asia Minor, it can be found in moist habitats in the high-altitude mountains including screes, stony areas, riverbanks, and stone walls; whereas in Russia, the species occurs in moist habitats within steppe and semi-desert areas, and it has also been found in closed-canopy mature forests [63,64,65,66].

In respect of the distribution maps constructed from IDW interpolation, *C. leucodon* is the only species that demonstrates two distinct abundance hotspots, one in eastern Thessaly plains and one in the western part (Figure 4). On the contrary, *S. etruscus* and *C. gueldenstaedtii* demonstrate their abundance hotspots in the southern and south-eastern parts of Thessaly plains, respectively (Figure 4). Comparing the shrews’ distribution patterns in Thessaly plains with the four species of voles in the same region [6], the most abundant vole species in Thessaly *Microtus hartingi* and *Microtus levis* appear with higher abundances in north-eastern and northern areas of Thessaly plains. Apparently, none of the three shrew species is present with high numbers in the areas where the dominant and abundant *M. hartingi* and *M. levis* present their higher abundances, and that could bring into the discussion the possibility of mutual exclusion due to competition and dominance. On the other hand, the significant models in all cases (*except C. leucodon*) denote a clear and strong association of shrew and vole species with concrete environmental gradients of soil types, soil texture, land uses, and crop types, creating a clear context of the underlying environmental forces shaping small-mammal distribution patterns in Thessaly. It must be pinpointed that in both cases of shrew species (*C. gueldenstaedtii* and *S. etruscus*) and vole species (*M. hartingi*, *M. levis, Microtus thomasi,* and *Cricetulus migratorius*), soil types and soil characteristics represent a major environmental gradient in small-mammal distribution, other than habitat types, land uses, and crops in agricultural land.

## 5. Conclusions

This is the first study in Greece that used the Barn owl diet as a proxy analysis with representative samples from a spatial extension of more than 3000 sq. km. in the major agricultural ecosystem of central Greece, Thessaly, which analyzed more than 10,000 pellets and more than 29,000 identified prey items. Out of three different shrew species in Thessaly plains, *C. gueldenstaedtii* and *S. etruscus* demonstrated a strong association with heavy argillaceous-clay soils and Vertisol soil types, which mainly defined their distribution patterns, in addition to non-irrigated land and non-intensive cultivated plots, which partially affected the presence of *S. etruscus* as well. *C. leucodon* demonstrated no attachment to any of the recorded environmental gradients, and its distribution pattern is of a generalist species with habitat plasticity, most possibly shaped by existing resources and intra/inter-specific competition. *C. leucodon* presents two abundance hotspots in Thessaly (east and west), whereas *C. gueldenstaedtii* and *S. etruscus* occupy, respectively, the south-eastern and southern areas of Thessaly plains. *C. gueldenstaedtii* was the most abundant shrew species in Thessaly plains and the second-most abundant small mammal in the area. We suggest incorporating if possible the different levels of soil texture and distinct soil types in future studies as part of the predictor datasets, in order to explore their effect as possible major drivers of the distribution patterns, habitat suitability, and abundance responses of shrews and, in general, of small mammals, in agricultural plains. We highlight the deep insights that can be gained in terms of small-mammal distribution and response patterns through the spectrum of Barn owl diet analysis on broad geographical scales.

## Figures and Tables

**Table 1:** Predictor variables calculated within the 2 km buffer around each Barn owl breeding site where pellets were collected.

Predictor Variables	Composition
Heavily Arable Land	Cereal Crops (wheat, barley, oat) Industrial Cultivations (cotton, tobacco, sugar beets)
Medium/Light Arable Land	Melon fields, Vegetables
Non Arable Land	Tree Cultivations, Vineyards
Other Land Uses	Fallow Land, Hills, Grassland, Pastures, Urban areas
Heavily Irrigated Land	Industrial Cultivations, Melon Fields, Vegetables
Medium/Lightly Irrigated Land	Cereals, Tree Cultivations, Vineyards
Non-Irrigated Land	remaining area after extracting the irrigated areas from total agricultural land
Alfisol Soil Type	-
Entisol Soil Type	-
Inceptisol Soil Type	-
Molllisol Soil Type	-
Vertisol Soil Type	-
Light Soils	Sandy Clay granulometric texture in the first 25 cm from soil surface/large particles and pore spaces
Heavy Soils	Argillaceous Clay granulometric texture in the first 25 cm from soil surface/fine clay particles difficult to manage but fertile when treated
River Length	linear measurement of rivers within each plot
Road Length	linear measurement of roads within each plot

**Table 2:** Indirect gradient analysis (PCA) taking into account only the variability in “species” matrix (dependent variables), and direct gradient analysis (RDA) taking into account the variability in both “species” and “environmental” (independent variables) matrix.

Principal Component Analysis (PCA)				
Axes	1	2	3	4
Eigenvalues	0.5313	0.2125	0.1044	0.0470
Cumulative percentage variance of species data	53.13	74.37	84.82	89.51
**Redundancy Analysis (RDA)**				
Axes	1	2	3	4
Eigenvalues	0.2299	0.0938	0.0397	0.0068
Explained variation (cumulative)	22.99	32.37	36.34	37.02
Pseudo-canonical correlation	0.6666	0.6582	0.6141	0.4943
Explained fitted variation (cumulative)	60.30	84.88	95.29	97.07

**Table 3:** Forward selection results on the initial predictor dataset of 16 variables. A total of 7 variables were included in the new explanatory dataset. Variables are ranked in order to explain total variation according to their significance. The percentage contribution for each environmental gradient is demonstrated to explain the fitted variation of the constrained model (RDA) during forward selection, on the procedure of selecting each variable in the model. Column “Explains %” represents the percentage of the total variation. Column “Contribution %” relates the contribution of each predictor to the explanatory power of the whole set of explanatory variables. Column “Pseudo-F” is the ratio between the eigenvalue of the single constrained (canonical) axis, which the term would define, and the average of the eigenvalues of the unconstrained (residual) axes. Column “*p* values” demonstrates significance tests run for each predictor variable and its effect on the model.

Predictor Variable	Explains %	Contribution 100%	Pseudo-F	*p* Values
Non-irrigated Land	11.4	30	15.7	0.0099
Other Land	4.2	10.9	6.0	0.0099
Vertisol Soil	4.7	12.4	7.1	0.0099
Heavy Soil	4.4	11.4	6.9	0.0099
Heavy Arable Land	2.1	5.4	3.3	0.0297
Light Soil	1.2	3.2	2.0	0.0351
Entisol	0.3	0.8	0.5	0.0459

**Table 4:** Response of shrew species to distinct predictor gradients. The “Best fit” model was selected based on Akaike criterion (AIC) through GLMs. The response variables that were rejected through the “null model” hypothesis did not fit any model and are not excluded from the table. Significant *p*-values are noted as * *p* < 0.05; ** *p* < 0.01; *** *p* < 0.001; **** *p* < 0.0001; ***** *p* < 0.00001. R2 (%) is a measurement of the actual explained variation, similar to determination coefficients in classical regression. Here, it is calculated as the ratio of deviance that is explained by the fitted model and the deviance of the null model multiplied by 100. Both the *p* estimate of the type I error rate and F test statistic correspond to an overall parametric test of the selected model against the null model, which pools the effect of both predictors when two predictors were present.

			Model Selection		GLM Results	
	R2 (%)	AIC	b_0_ + b_1_X	b_0_ + b_1_X + b_2_X^2^	F	*p*
Heavy Arable Land						
*Crocidura leucodon*	5.8	530.73		√	3.7	*
*Crocidura gueldenstaedtii*	7.9	182.27	√		10.5	**
*Suncus etruscus*	2.8	637.42	√		3.5	0.06423
Other Land						
*Crocidura leucodon*	5.5	530.38		√	3.5	*
*Crocidura gueldenstaedtii*	4.3	177.50	√		5.5	*
*Suncus etruscus*	1.9	636.26	√		2.3	0.13039
Non-irrigated Land						
*Crocidura leucodon*	4.7	529.25		√	3.0	0.05608
*Suncus etruscus*	7.5	643.60	√		9.9	*
Heavy Soil						
*Crocidura gueldenstaedtii*	17.9	194.38		√	13.2	*****
*Suncus etruscus*	6.4	640.03		√	4.2	*
Light Soil						
*Crocidura gueldenstaedtii*	19.8	197.34		√	15.0	*****
*Suncus etruscus*	7.6	641.58		√	5.0	**
Vertisol						
*Crocidura gueldenstaedtii*	20.4	200.32	√		31.2	*****
*Suncus etruscus*	18.5	659.30	√		27.7	*****
Entisol						
*Crocidura leucodon*	5.3	530.08		√	3.4	*
*Crocidura gueldenstaedtii*	9.8	182.71		√	6.6	**
*Suncus etruscus*	7.7	643.80	√		10.1	**

## Data Availability

Data is contained within the article.
